# The relationship of bottle feeding and other sucking behaviors with speech disorder in Patagonian preschoolers

**DOI:** 10.1186/1471-2431-9-66

**Published:** 2009-10-21

**Authors:** Clarita Barbosa, Sandra Vasquez, Mary A Parada, Juan Carlos Velez Gonzalez, Chanaye Jackson, N David Yanez, Bizu Gelaye, Annette L Fitzpatrick

**Affiliations:** 1 Corporacion de Rehabilitacion Club De Leones Cruz Del Sur, Punta Arenas, Chile; 2 Multidisciplinary International Research Training Program, University of Washington, School of Public Health, Seattle, Washington, USA; 3 Department of Biostatistics, University of Washington, School of Public Health, Seattle, Washington, USA; 4 Departments of Epidemiology and Global Health, University of Washington, School of Public Health, Seattle, Washington, USA

## Abstract

**Background:**

Previous studies have shown that children's nonnutritive sucking habits may lead to delayed development of their oral anatomy and functioning. However, these findings were inconsistent. We investigated associations between use of bottles, pacifiers, and other sucking behaviors with speech disorders in children attending three preschools in Punta Arenas (Patagonia), Chile.

**Methods:**

Information on infant feeding and sucking behaviors, age starting and stopping breast- and bottle-feeding, pacifier use, and other sucking behaviors, was collected from self-administered questionnaires completed by parents. Evaluation of speech problems was conducted at preschools with subsequent scoring by a licensed speech pathologist using age-normative standards.

**Results:**

A total of 128 three- to five-year olds were assessed, 46% girls and 54% boys. Children were breastfed for an average of 25.2 (SD 9.6) months and used a bottle 24.4 (SD 15.2) months. Fifty-three children (41.7%) had or currently used a pacifier for an average of 11.4 (SD 17.3) months; 23 children (18.3%) were reported to have sucked their fingers. Delayed use of a bottle until after 9 months appeared to be protective for subsequent speech disorders. There was less than a one-third lower relative odds of subsequent speech disorders for children with a delayed use of a bottle compared to children without a delayed use of a bottle (OR: 0.32, 95% CI: 0.10-0.98). A three-fold increase in relative odds of speech disorder was found for finger-sucking behavior (OR: 2.99, 95% CI: 1.10-8.00) and for use of a pacifier for 3 or more years (OR: 3.42, 95% CI: 1.08-10.81).

**Conclusion:**

The results suggest extended use of sucking outside of breastfeeding may have detrimental effects on speech development in young children.

## Background

It has been suggested that children in the Chilean Patagonia use milk bottles and pacifiers far beyond recommendations of health personnel. Of primary importance is answering the question, what type of feeding, breast or bottle, is better for oral cavity architecture and the influence on the acquisition of early speech. The development of oral motor structures is reflected on craniofacial development and dentition [1]. To identify potential risk factors for speech disorders in children, there is a need to better understand the association between early life feeding and sucking behaviors and subsequent speech development.

The relationships between children's sucking habits and the impact on the development of their oral anatomy and functioning have been described in the literature. Agurto *et al *studied 1,110 Chilean children between the ages of 3 to 6 years of age. They reported bad oral habits were associated with development of dentomaxilar anomalies [2]. Linder and Modeer studied 76 four year old children to investigate the relationship between sucking habits (dummy or fingers) and dental characteristics in children with unilateral cross bite. The results indicated that duration and intensity of sucking habits may adversely influence dental characteristics by reducing the transverse width of the maxillary arch [3].

Duncan *et al *studied a cohort of 867 children using a family questionnaire on sucking habits at 15, 24, and 36 months of age and a dental examination at 31, 43, and 61 months of age. The results indicated that at 15 months, 63% of children had a sucking habit, 38% used just a dummy and 23% used a digit. By 36 months, sucking had reduced to 40% with similar prevalence of dummy and digit sucking. Both habits had effects on developing dentition, most notably on upper labial segment alignment and the development of anterior open bites and posterior cross bites [4]. In one study [5] involving 108 children, a significant association between children who were bottle fed and presence of anteroposterior malocclusion was reported. Breastfeeding was also found to decrease the risk of getting this type of malocclusion. The investigators noted that when bottle feeding occurs, only the buccinator muscles and the orbicular muscle(s) of the mouth are exerted without stimulating other muscles. They concluded sucking only during breastfeeding promotes correct muscle activity, and thus proper development of the oral motor structures [5].

Broad performed a study in 1972 in Putaruru, New Zealand that examined the effects of infant feeding on speech quality [6]. Broad investigated clarity of articulation, tonal quality, confidence, and reading ability in 5 and 6 year old children. There was a significant association between clarity of speech and breastfed males but not females, and breastfeeding was associated with improved tonal quality and reading ability of both males and females [6]. Breastfeeding has been found to be beneficial in other studies of linguistic and cognitive development [7]. The development of coordinated breathing, chewing, swallowing and speech articulation has also been shown to be associated with breastfeeding. It is believed that breastfeeding promotes mobility, strength, and posture of the speech organs. Such speech organs include: lips, tongue, maxilla, mandible, cheeks, soft palate, hard palate, dental arch, floor of mouth, and more. In order for speech development to occur, the child must suck with consistent rhythm and strength. Movements while sucking can cause absorption of the sucking pads and growth of the mandible. As a result, the intra-oral space increases [1]. Moreover, studies have shown that breastfeeding protects normal dentition [8-10].

Fox *et al *in their study of German children with speech-disorders reported a significantly higher incidence of bottle and pacifier use compared to normal children [11]. Children of industrialized western countries are more likely to use pacifiers and to feed using a bottle than children in developing countries. Over the last few decades, use of bottles and pacifiers has increased approximately 75% to 79% in the West [12-14]. In non-industrialized countries such as Tanzania and Zimbabwe, pacifier use and finger sucking are less common or non-existent [14]. This has also been found in families with lower social economic status. A study conducted in Santiago, Chile by Olguin and Quintana reported 28% of breastfed and 52% of non-breastfed children used pacifiers [15]. They also found that mothers (88% of the time) were more likely to use of pacifiers without a specific reason for their use [15]. It is reasonable to conclude that whether a child is breast or bottle fed depends on both cultural and economic factors.

From the above it is apparent that feeding-sucking behaviors and speech-oral anatomy development have positive and negative impacts on speech. In the current study we intended to move beyond assessment of oral musculature to the speech disorder that may impair communication and literacy [16]. We describe an observational study designed to evaluate risk factors among pre-school Chilean Patagonia children focusing on past and present sucking behaviors as reported by their parents. We also sought to see the extent to which early feeding and sucking patterns might influence speech disorders.

## Methods

Data were collected on 128 children aged 37 to 70 months old attending three local public kindergartens in Punta Arenas (Patagonia), Chile, during the school years of 2006 and 2007. Information was gathered utilizing parent questionnaires, child speech evaluations and physical examinations of the children's mouths conducted by a pediatrician. Parental informed consent and assent from the participating children were received prior to conduct of the study. This project was reviewed by and received approval from the local Institutional Review Board governing this research (Centro de Rehabilitacion Club de Leones Cruz del Sur, Punta Arenas, Chile). Before analysis, personal identifiers were removed from each data set. The Human Subjects Division of the University of Washington, USA granted approval to use the de-identified and anonymised data set for analysis.

### Parent Questionnaires

Parent questionnaires [Additional File 1] consisted of 79 questions intended to collect information on each child's feeding history, demographics, and social economic status. To investigate the effect of oral development from feeding, the questionnaire asked parents to answer the following questions: whether or not the child drank from a bottle and if yes, how often; in what position, and when the child stopped bottle feeding (if not still using one). Similar questions were asked about use of a pacifier, use of a security blanket (it is often common for children in Chile to suck the blanket while going to sleep), and whether the child sucked their fingers. Parental (mother and father) education, income, and parental perceptions about the child's ability to communicate were also surveyed.

### Speech Evaluation

A standard phonological evaluation used by Chilean speech therapists, TEPROSIF (test to evaluate simplified phonological processes) was utilized to determine the type and number of errors in the child-age related phonological processes. TEPROSIF is based on the natural phonology theory from the classical work of Stampe [17] and Ingram [18]. This theory proposes that during development, children produce words in a simplified manner using a group of simplification strategies known as phonological processes. The TEPROSIF test was validated among 620 normal Chilean children between the ages of 3 and 7 years old [19]. The validation study was conducted in 5 different regions of the country. The study findings indicated that the TEPROSIF test had high degree of reliability (Cronbach alpha = 0.9). Another study done among children with specific language disorders and a control group showed that children with speech language disorders produced phonological processes more often than the normal controls (p < 0.005) [19].

To perform this test, an evaluator first shows a child a series of black and white drawings from a test booklet. Then the examiner tells him/her a standardized phrase that includes the name of the figure and the child is asked to imitate the production. If the child does not complete the phrase, the examiner repeats the name of the figure and asks the child to repeat it. The responses are used to determine child's ability to produce particular speech sounds. These responses are written down, phonetically transcribed by a licensed speech therapist. Common errors are determined including changes of syllable structure, substitution, and assimilation. Categories scores were determined using procedures previously described by Maggiolo and Pavez [16]. Those with mean for age +/- 1 standard deviations (SD) were categorized as normal; those with less than -1SD were grouped as below normal, and those with greater than 1SD were categorized as above normal.

### Statistical Analysis

Descriptive statistics were calculated using cross-tabulations for categorical variables and grouped means and standard deviations for continuously measured variables. In Tables 1 and 2, chi-square tests were used to compare the age categories and categorical characteristics; regression analysis was used to compare continuous characteristics to age. In Table 3, chi-square tests were used to compare categorical characteristics to the TEPROSIF classifications. The p-values are omnibus tests of association between the two variables. Multivarible logistic regression was used to investigate the associations between potential risk factors and speech disorder outcomes. Both unadjusted models and adjusted models (adjusting for gender and age) were fitted to obtain estimated odds ratios (ORs) and 95% confidence intervals (CIs). Wald test statistics were used in all hypothesis tests. All p-values are two-sided. These analyses were conducted using the SPSS (version 13.0) statistical package.

**Table 1:** Selected characteristics of 128 children by age in Punta Arenas, 2006-2007

	**Age Category**				
**Characteristic**	**3**	**4**	**5**	**Total**	
**(N)**	**58**	**49**	**21**	**128**	
	**N (%) or** **Mean (SD)**	**N (%) or** **Mean (SD)**	**N (%) or** **Mean (SD)**	**N (%) or** **Mean (SD)**	**p**

**Sex**					
Female	30 (51.7)	19 (38.8)	10 (47.6)	59 (46.1)	.40
Male	28 (48.3)	30 (61.2)	11 (52.4)	69 (53.9)	
**Mothers age at birth**					
15-20	15 (25.9)	12 (25.0)	6 (28.6)	33 (26.0)	.33
21-25	15 (25.9)	21 (43.8)	6 (28.6)	42 (33.1)	
≥ 26	28 (48.3)	15 (31.3)	9 (42.9)	52 (40.9)	
**Mothers Education**					
Middle School	13 (22.4)	12 (24.5)	7 (33.3)	32 (25.0)	.80
High School	28 (48.3)	23 (46.9)	7 (33.3)	58 (45.3)	
College	17 (29.3)	14 (28.6)	7 (33.3)	38 (29.7)	
**Fathers Education**					
Middle School	8 (15.1)	5 (11.1)	2 (10.5)	15 (12.8)	.34
High School	35 (66.0)	23 (51.1)	12 (63.2)	70 (59.8)	
College	10 (18.9)	17 (37.8)	5 (26.3)	32 (27.4)	
**Family Income**					
Less than 180,000 p	14 (24.1)	11 (23.4)	6 (30.0)	31 (24.8)	.50
180,000 - 250,000 p	14 (24.1)	11 (23.4)	5 (25.0)	30 (24.0)	
250,000 - 340,000 p	19 (32.8)	9 (19.1)	6 (30.0)	34 (27.2)	
More than 340,000 p	11 (19.0)	16 (34.0)	3 (15.0)	30 (24.0)	
**Gestation**					
Normal (38-40)	42 (72.4)	32 (66.7)	17 (81.0)	91 (71.7)	.30
Under Normal	6 (10.3)	11 (22.9)	2 (9.5)	19 (15.0)	
Above Normal	10 (17.2)	5 (10.4)	2 (9.5)	17 (13.4)	
**Birth weight**					
Normal	36 (62.1)	29 (59.2)	12 (57.1)	77 (60.2)	.25
Underweight	7 (12.1)	12 (24.5)	2 (9.5)	21 (16.4)	
Overweight	15 (25.9)	8 (16.3)	7 (33.3)	30 (23.4)	
**Insurance**					
Fonasa	53 (93.0)	44 (89.9)	20 (95.2)	117 (92.1)	.70
Private	4 (7.0)	5 (10.2)	1 (4.8)	10 (7.9)	
**Hours of TV per day**					
Less than 3	28 (48.3)	26 (53.1)	8 (38.1)	62 (48.4)	.74
3-5 hours	28 (48.3)	20 (40.8)	12 (57.1)	60 (46.9)	
More than 5 hrs	2 (3.4)	3 (6.1)	1 (4.8)	6 (4.7)	

**Table 2:** Sucking behaviors of children ages 3 to 5 in Punta Arenas, Chile 2006-2007

	**Age Category**				
**Characteristic**	**3**	**4**	**5**	**Total**	
**(N)**	**58**	**49**	**21**	**128**	
	**N (%) or** **Mean (SD)**	**N (%) or** **Mean (SD)**	**N (%) or** **Mean (SD)**	**N (%) or** **Mean (SD)**	**p**

**Bottle fed**					
No	2 (3.5)	3 (6.1)	2 (9.5)	7 (5.5)	.57
Yes	55 (96.5)	46 (93.9)	19 (90.5)	120 (94.5)	
**Time bottle feeding**					
Less than 18 mths	9 (16.4)	8 (17.8)	1 (5.6)	18 (15.3)	.26
18 to 36 mths	24 (43.6)	12 (26.7)	9 (50.0)	45 (38.1)	
More than 36 mths	22 (40.0)	25 (55.6)	8 (44.4)	55 (46.6)	
**Breastfed**					
No	3 (5.3)	2 (4.1)	0 (0.0)	5 (3.9)	.57
Yes	54 (94.7)	47 (95.9)	21 (100.0)	122 (96.1)	
**Time breastfeeding**					
Less than 6 mths	16 (27.6)	17 (34.7)	5 (23.8)	38 (29.7)	.83
6 to 12 mths	24 (41.4)	19 (38.8)	8 (38.1)	51 (39.8)	
More than 12 mths	18 (31.0)	13 (26.5)	8 (38.1)	39 (30.5)	
**Use of pacifier**					
No	33 (57.9)	28 (57.1)	13 (61.9)	74 (58.3)	.93
Yes	24 (42.1)	21 (42.8)	8 (38.1)	53 (41.7)	
**Time with pacifier**					
2 to 12 mths	6 (25.0)	5 (25.0)	1 (12.5)	12 (23.1)	.33
12 to 24 mths	6 (25.0)	6 (30.0)	0 (0)	12 (23.1)	
24 to 36 mths	3 (12.5)	5 (25.0)	2 (25.0)	10 (19.2)	
Greater than 36 mths	9 (37.5)	4 (20.0)	5 (62.5)	18 (34.6)	
**Sucked fingers**					
No	46 (80.7)	41 (83.7)	16 (80.0)	103 (81.7)	.90
Yes	11 (19.3)	8 (16.3)	4 (20.0)	23 (18.3)	
**Time sucking finger**					
0 to 12 mths	6 (54.5)	0 (0.0)	1 (25.0)	7 (41.2)	.52
12 to 30 mths	3 (27.3)	1 (50.0)	1 (25.0)	5 (29.4)	
> 30 mths	2 (18.3)	1 (50.0)	2 (50.0)	5 (29.4)	

**Table 3:** Sucking behaviors by phonological processes categorizations from the TEPROSIF examination for 128 Children ages 3 to 5 in Punta Arenas, 2006-2007

	**TEPROSIF CLASSIFICATION**				
**Characteristic**	**Below normal**	**Normal**	**Above Normal**	**Total**	
**(N)**	**61**	**42**	**25**	**128**	
	**N (%) or** **Mean (SD)**	**N (%) or** **Mean (SD)**	**N (%) or** **Mean (SD)**	**N (%) or** **Mean (SD)**	**p**

**Bottle fed**					
No	4 (6.6)	1 (2.4)	2 (8.0)	7 (5.5)	.54
Yes	57 (93.4)	41 (97.6)	23 (92.0)	121 (94.5)	
**Time bottle feeding**					
Less than 18 mths	9 (15.8)	7 (17.9)	2 (9.1)	18 (15.3)	.69
18 to 36 mths	19 (33.3)	17 (43.6)	9 (40.9)	45 (38.1)	
More than 36 mths	29 (50.9)	15 (38.5)	11 (50.0)	55 (46.6)	
**Breastfed**					
No	3 (4.9)	2 (4.8)	0 (0.0)	5 (3.9)	.53
Yes	58 (95.1)	40 (95.2)	25 (100.0)	123 (96.1)	
**Time breastfeeding**					
Less than 6 mths	22 (36.1)	13 (31.0)	3 (12.0)	38 (29.7)	.17
6 to 12 mths	23 (37.7)	14 (33.3)	14 (56.0)	51 (39.8)	
More than 12 mths	16 (26.2)	15 (35.7)	8 (32.0)	39 (30.5)	
**Use of pacifier**					
No	33 (54.1)	24 (57.1)	17 (68.0)	74 (57.8)	.49
Yes	28 (45.9)	18 (42.9)	8 (32.0)	54 (42.2)	
**Time with pacifier**					
2 to 12 mths	7 (25.9)	3 (17.6)	2 (25.0)	12 (23.1)	.23
12 to 24 mths	3 (11.1)	7 (41.2)	2 (25.0)	12 (23.1)	
24 to 36 mths	4 (14.8)	4 (23.5)	2 (25.0)	10 (19.2)	
Greater than 36 mths	13 (48.1)	3 (17.6)	2 (25.0)	18 (34.6)	
**Sucked fingers**					
No	45 (73.8)	35 (83.3)	24 (100.0)	104 (81.9)	.02
Yes	16 (26.6)	7 (16.7)	0 (0.00)	23 (18.1)	
**Time sucking finger**					
0 to 12 mths	3 (27.3)	4 (66.7)	0 (0.00)	7 (41.2)	.29
12 to 30 mths	4 (36.4)	1 (16.7)	0 (0.00)	5 (29.4)	
> 30 mths	4 (36.4)	1 (16.7)	0 (0.00)	5 (29.4)	

## Results

### Descriptive Analyses

Table 1 provides a summary of selected characteristics and children's ages in our study sample in this study. Of 128 children total, there were 58 three year olds (45%), 49 four year olds (31%) and 21 five year olds (13%). Twenty-six percent of mothers were younger than 20 years, approximately 50% of the mothers had high school education, and 30% were college educated. Approximately 75% of mothers had a normal gestation period, although 19 births (15%) occurred in less than 38 weeks of gestation. Approximately 16% babies had low birth weight (< 2500 grams).

In Table 2, we present summaries on breastfeeding and other sucking behaviors versus children's age. Five children (4%) were not breastfed, 30% were breastfed for more than one year. Almost all (85%) children were bottle fed more than 18 months; almost half of children were bottle fed more than three years (47%). Forty two percent of the children used a pacifier and approximately 33% of these children used them for more than 3 years. Only 23 children (18.3%) were reported to have sucked their fingers for comfort.

### Association with phonological processes

Table 3 shows sucking behavior according to the evaluated level of phonological processes. In these bivariate summaries only one behavior, having ever sucked their finger, was significantly associated with the three speech processing classifications (p = .02). Several other variables showed higher percentages of children with below normal speech processing classifications and high levels of sucking behaviors. These associations, however, were not statistically significant. Children with below normal occurrence of speech phonological processes were breastfed for a shorter period of time; only 26% were breastfed for 12 months or longer, compared to 35.7% and 32.0% with normal or above normal phonological processes respectively. More than twice as many children with below normal phonological processes used a pacifier for more than three years compared to those without speech problems.

Logistic regression was used to evaluate associations between feeding/sucking behaviors and the level of phonological processes categorized as below normal versus normal. The unadjusted and adjusted (gender and age) results are shown in Table 4. The results indicate children born pre-term had an increased risk of developmental problems with speech; children with gestational age less than 38 weeks had three times higher odds of having an abnormal score on the TEPROSIF compared to those of normal gestation group (OR: 3.27, 95% CI: 1.0 - 10.2). Adjusted for age and gender, delayed use of a bottle until after 9 months appears to be protective from subsequent speech disorders by less than one-third relative odds (OR: 0.32, 95% CI: 0.10-0.98). A three-fold increase in relative odds of speech disorder was found with any finger-sucking behavior (OR: 2.99, 95% CI: 1.10 - 8.00). Those who used a pacifier for 3 or more years were so found to have a three-fold an increased relative odds of speech disorders (OR: 3.4, 95% CI: 1.08-10.81).

**Table 4:** Associations between below normal scores on the TEPROSIF evaluation and early-life feeding or sucking behaviors using logistic regression.

**Risk Factor**	**Unadjusted** **OR (95% CI)**	**p**	**Adjusted^1^** **OR (95% CI)**	**P**
**Gestation**				
Normal (38 - 40 wks)	1.0 (REF)	.105	1.0 (REF)	.09
Below normal	3.17 (1.0, 9.6)	.042	3.27 (1.0, 10.2)	.04
Above normal	.854 (.30, 2.4)	.768	.745 (.25, 2.18)	.59
**Age started bottle feeding**				
Less than 3 mths	1.0 (REF)	.151	1.0 (REF)	.13
3 to 9 mths	.617 (.27, 1.4)	.247	.601 (.26, 1.4)	.23
More than 9 mths	.339 (.11, 1.0)	.055	.315 (.10, .98)	.05
**Bottle fed**				
No	1.0 (REF)	.621	1.0 (REF)	.48
Yes	.679 (.15, 3.2)	.621	.568 (.1, 2.8)	
**Time bottle feeding**				
Less than 18 mths	1.0 (REF)	.521	1.0 (REF)	.59
18 to 36 mths	.731 (.24, 2.2)	.575	.788 (.26, 2.4)	.68
More than 36 mths	1.16 (.40, 3.4)	.785	1.21 (.41, 3.6)	.73
**Breastfed**				
No	1.0 (REF)	.588	1.0 (REF)	.63
Yes	.604 (.10, 3.7)	.588	.637 (.10, 4.0)	
**Time breastfeeding**				
Less than 6 mths	1.0 (REF)	.242	1.0 (REF)	.28
6 to 12 mths	.560 (.24, 1.3)	.185	.559 (.24, 1.33)	.19
More than 12 mths	.474 (.19, 1.2)	.110	.496 (.20, 1.3)	.14
**Use of pacifier**			1.0 (REF)	
No	1.0 (REF)	.360	1.0 (REF)	.39
Yes	1.39 (.69, 2.8)	.360	1.37 (.67, 2.8)	
**Time with pacifier**				
No use	1.0 (REF)	.07	1.0 (REF)	.08
Less than one year	1.74 (.51, 5.98)	.38	1.54 (.44, 5.45)	.50
1-3 years	.58 (.21, 1.59)	.29	.58 (.21, 1.61)	.29
Greater than 3 years	3.23 (1.04, 9.99)	.04	3.42 (1.08, 10.81)	.04
**Sucked fingers**				
No	1.0 (REF)	.029	1.0 (REF)	.03
Yes	2.95 (1.1, 7.8)	.029	2.99 (1.1, 8.0)	
**Time sucking finger**				
0 to 12 mths	1.0 (REF)	.314	1.0 (REF)	.21
12 to 30 mths	5.33 (.34, 75.8)	.216	12.3 (.45, 343.5)	.14
> 30 mths	5.33 (.34, 75.8)	.216	14.5 (.48, 442.0)	.12

Odds ratios (OR) and 95% confidence intervals (CI) are shown

^1^Adjusted for gender and age

## Discussion

Results of this study indicate that finger sucking behaviors and prolonged use of a pacifier for 3 years or more may be detrimental to optimal speech development in young children. There was less than a one-third lower relative odds of subsequent speech disorders for children with a delayed use of a bottle compared to children without a delayed use of a bottle (OR: 0.32, 95% CI: 0.10-0.98). A three-fold increase in relative odds of speech disorder was found for finger-sucking behavior (OR: 2.99, 95% CI: 1.10, 8.00) and for use of a pacifier for 3 or more years (OR: 3.42, 95% CI: 1.08, 10.81).

While our study findings indicate that habits of longer durations (longer than three years) may provide the greatest risk of speech disorders, others have found that nonnutritive sucking habits of shorter durations may affect oral development as well. Warren *et al *[10] studied dental arch and occlusal conditions of 4 to 5 years old children with a variety of different nonnutritive sucking habit durations. They found that children with nonnutritive sucking habits past the age of 48 months, compared to children with a shorter duration of nonnutritive sucking habits, were more likely to have narrower maxillary arch widths, greater overjet, higher prevalence of open bite, and posterior crossbite. They concluded that while habits continuing past 48 months produced the greatest changes in dental arch and occlusal characteristics, there are also detectable differences between children that have had shorter sucking durations and minimal sucking durations [20]. It has been proposed, however, that these effects may be reversible. Verrastro *et al *evaluated occlusal and orafacial myofunctional characteristics of twenty seven 3 to 5 year old-children. They reported that removal of pacifier sucking habit was significantly associated with a reduction of 2 mm on anterior bite (P < .001), an improvement of lip posture (P = .03), favored nasal breathing (P = . 008), and a reduction in the occurrence of tongue interposition while swallowing (P = .008) [21].

In Brazil, Tomita *et al *examined the effect of oral habits and speech problems on dental occlusion in a cross-sectional study of 2,139 children between the ages 3 to 5 years. They found that habit of sucking a pacifier was a greater risk factor for malocclusion (OR = 5.46) followed by habit of finger sucking (OR 1.54). They also reported found that speech did not show any influence in malocclusion occurrence [22].

Our study involved a number of strengths as well as limitations. Strengths included a larger sample size compared to other studies, the use of a standardized test to evaluate the children's speech and the use of a speech pathologist to score the tests. There are limitations to this study. First, due to the observational nature of the study design, confounding factors could adversely impact our findings. Second, the parent's survey data were collected by self-reports and there is the possibility of measurement error and recall biases. Measurement error tends to bias the observed results to the null and one might reasonably assume the associations would be stronger if more precisely measured variables were collected. It may also be a challenge to generalize these results to a wider population of children. At last, the fact that some of the sample children were born prematurely, being them at greater risk per se for speech delays.

## Conclusion

These results suggest that sucking habits such as pacifier use, finger sucking and bottle feeding are associated with speech disorders in preschool children. The age at which the child started bottle feeding was separated into three categories, less than three months, three to nine months, and more than nine months. Starting bottle feeding after 9 months was found to be better for the suppression of phonological processes, since it is protective against obtaining an abnormal classification on the Test for classifying these processes. Finger sucking, on the other hand, proved to be harmful to the development of these processes. This is reflected on the finding that children who suck their fingers were about three times more likely to obtain an abnormal classification on the TEPROSIF evaluation of simplified phonological processes. Pacifier use was also shown to negatively impact the development of speech alterations if used for more than three years while less was not found to be harmful. Although results of this study provide further evidence for the benefits of longer duration of breastfeeding of infants, they should be interpreted with caution as these data are observational. Further investigation of larger studies and clinical trials are needed to confirm these findings.

## Competing interests

The authors declare that they have no competing interests.

## Authors' contributions

CB, SV, and JCV conceived the study, participated in the design of the study, carried out data collection, and drafted the manuscript. MP and CJ participated in data analysis, interpretation and drafted the manuscript. AF supervised the study and the students for this project and led the analysis, participated in interpretation, drafting and critical review of the manuscript. NDY participated in data analysis, interpretation, contributed towards drafting and provided critical review of the manuscript. BG participated in critical review and format of the manuscript. All authors read and approved the final manuscript

## Pre-publication history

The pre-publication history for this paper can be accessed here:



## Supplementary Material

Additional file 1 **Questionnaire**. Questionnaire administered in the study. Click here for file
