# Supplementary material for: The relationship of bottle feeding and other sucking behaviors with speech disorder in Patagonian preschoolers
Source: BMC Pediatr. 2009 Oct 21;9:66. doi: 10.1186/1471-2431-9-66 (PMC2773774; doi:10.1186/1471-2431-9-66)
Supplement: Additional file 1 — Questionnaire. Questionnaire administered in the study. [file 1471-2431-9-66-S1.PDF]

Corporación de Rehabilitación "Cruz del Sur"

Investigación Efecto de la mamadera y el chupete en el desarrollo de los procesos fonológicos.

N°: ____________________ Fecha: _____________________

Nombre completo del niño(a):

____

1. Edad del niño(a): __________________
2. Fecha de nacimiento del niño(a): __________________
3. Edad de madre al nacimiento del niño(a): __________ años
4. ¿Tuvo control médico la madre durante el embarazo?: ____Sí ___No

   ¿En qué mes del embarazo inició el control medico?: _________ mes(es)
5. ¿Lugar de nacimiento del niño(a)?: ____________________________________
6. ¿Cuantas semanas de embarazo tenía la madre al momento del nacimiento del niño(a)?: __________________
7. ¿Cuál fue el peso del niño(a) al nacimiento?: __________ k.g.
8. ¿Estuvo hospitalizado el niño(a) durante el primer mes de vida?: ____Sí ___No

¿Por qué?: ____

9. ¿A que edad el niño(a) comenzó a caminar?: __________________
10. ¿A qué jardín infantil asiste el niño(a)?: ____________________________________
11. ¿Qué tan frecuente se enferma el niño(a) en un año?: ____Siempre ____A Veces ____ Nunca
12. ¿Cómo considera la salud del niño(a)? ____Buena ____Regular ____Mala
13. ¿Durante el primero año el niño(a) recibió suplemento de:

    Hierro? ____Sí ___No

    Vitamina A? ____Sí ___No

    Vitamina C? ____Sí ___No

    Vitamina D? ____Sí ___No
14. ¿Ha tenido el niño(a) alguna vez otitis media?: ____Sí ___No

    Si la respuesta es **Sí:** ___1 a 3 episodios ___3 a 10 episodios ___Más de 10

15. **Uso de Mamadera:**

- ¿Su hijo(a) usa, ha usado o usó mamadera?: ____Sí ___No
- ¿A qué edad a su hijo(a) se le inició la mamadera? ___________________
- ¿Cuántas mamaderas recibe su hijo(a)?: N°_____ en el Día N°_____ en la Noche
- ¿Posición de su hijo(a) al tomar la mamadera?: ____Acostado ____Sentado
- ¿A que edad le retiró a su hijo(a) la mamadera?: ___________________
- ¿Usa su hijo(a) mamadera en la actualidad?: ____Sí ____No ____ A veces

16. **Uso de Chupete:**

- ¿Su hijo(a) usa, ha usado o usó chupete?: ____Sí ___No
- ¿Qué edad tenía su hijo(a) al iniciar el chupete?: ___________________
- ¿Con que frecuencia usa o usó su hijo(a) el chupete?: ____Día ____Noche ____A veces
- ¿A que edad le retiró a su hijo(a) la chupete?: ___________________
- ¿Usa su hijo(a) chupete en la actualidad?: ____Sí ____No ____ A veces

17. **Uso de Tuto:**

- ¿Su hijo(a) usa, ha usado o usó tuto?: ____Sí ___No
- ¿A qué edad su hijo(a) empezó a usar el tuto?: ___________________
- ¿Con qué frecuencia su hijo(a) usó o usa el tuto?: ____Día ____Noche ____A veces
- ¿A qué edad le retiró a su hijo(a) el tuto?: ___________________
- ¿Usa su hijo(a) tuto en la actualidad?: ____Sí ____No ____ A veces

18. **Chupar el Dedo:**

- ¿Su hijo(a), chupa, ha chupado, o chupó dedo?: ____Sí ___No
- ¿A que edad su hijo(a) empezó a chuparse el dedo?: ___________________
- ¿Con que frecuencia su hijo(a) se chupa el dedo?: ____Día ____Noche ____A veces
- ¿A qué edad su hijo(a) dejó de chuparse el dedo?: ___________________
- ¿Su hijo(a) chupa dedo en la actualidad?: ____Sí ____No ____ A veces

19. **Lactancia Materna:**

- ¿Recibió su hijo(a) lactancia materna?: ____Sí ___No

- ¿Recibió su hijo(a) lactancia materna y relleno a la vez?: ____Sí ___No

- ¿Cuánto tiempo recibió su hijo(a) lactancia materna?: ___________________

- ¿Tuvo grandes problemas su hijo(a) para adaptarse o mantener la lactancia materna que hicieron necesario que su hijo(a) empezara a tomar relleno?: ____Sí ___No

- ¿Tuvo problemas su hijo(a) para succionar el pecho?: ____Sí ___No

- ¿Qué edad tenía su hijo(a) cuándo se le suspendió la lactancia materna?: ___________________

- ¿Porqué razón usted le suspendió a su hijo(a) la lactancia materna?:
  - No tenía más leche: ____Sí ___No
  - La madre tenía que tomar un medicamento: ____Sí ___No
  - La madre tenía una enfermedad no compatible con la lactancia: ____Sí ___No
  - Otra razón: ____Sí ___No

    Cuál: ____

20. **Audición, Comprensión y Emisión del Lenguaje:**
Por favor marca la caja.

	Siempre	A Veces	Nunca
¿Considera usted que su hijo(a) oye bien?			
¿Considera usted que su hijo(a) entiende y comprende todo lo que dicen?			
¿Cuándo su hijo(a) habla con otros niños o personas adultas diferentes a su familia, usted considera que le entienden?			
¿Para que a su hijo(a) le entienden lo que habla usted necesita interpretarle a los demás lo que su niño quiere decir?			
¿Presenta su hijo(a) dificultad para pronunciar algunas letras, que impida que los demás le entiendan cuando el/la habla?			
¿Considera usted que su hijo(a) entiende lo que se le dice y es capaz de seguir instrucciones?			
¿Ha sido diagnosticado su hijo(a), alguna vez con alguna alteración del lenguaje en los controles de niño sano?			

¿Considera usted que su hijo(a), tiene un nivel de lenguaje acorde a su edad?			

21. ¿Con quien vive el niño(a)?: ____________________________

22. Ocupación del padre: ____________________________

¿Cuánto dinero gana en un mes?: ____________________________

23. Ocupación de la madre: ____________________________

¿Cuánto dinero gana en un mes?: ____________________________

24. Ocupación del cuidador: ____________________________

¿Cuánto dinero gana en un mes?: ____________________________

Marque su respuesta con un círculo.

Por Ejemplo
Nivel de escolaridad de la madre:

¿Básica? **¿Media?** **¿Técnica?** **¿Universitaria?**

Completa / Incompleta

25. **Nivel de escolaridad de la madre:**

¿Básica? ¿Media? ¿Técnica? ¿Universitaria?

Completa / Incompleta

26. **Nivel de escolaridad del padre:**

¿Básica? ¿Media? ¿Técnica? ¿Universitaria?

Completa / Incompleta

27. **Nivel de escolaridad del cuidador**

¿Básica? ¿Media? ¿Técnica? ¿Universitaria?

Completa / Incompleta

Marque su respuesta con un círculo
28. Previsión:

¿Fonasa? ¿Isapre? ¿Particular?

29. ¿Es su casa propia?: ____Sí ___No

30. ¿Cuántas horas en el día mira su niño(a) televisión?: ________ horas por día

31. ¿Tiene o ha tenido televisión por cable?: ____Sí ___No

32. ¿En este momento en qué ciudad está su lugar de domicilio? __________________________

33. Dirección: ____

Muchas gracias para responder nuestro cuestionario. Si tiene cualquier pregunta por favor contactar a Dra. Clarita Barbosa o a Sandra Vásquez, fonoaudióloga en el Centro de Rehabilitación Club de Leones “Cruz del Sur”
